# Time-of-Day-Dependent Effects of Bromocriptine to Ameliorate Vascular Pathology and Metabolic Syndrome in SHR Rats Held on High Fat Diet

**DOI:** 10.3390/ijms22116142

**Published:** 2021-06-07

**Authors:** Michael Ezrokhi, Yahong Zhang, Shuqin Luo, Anthony H. Cincotta

**Affiliations:** VeroScience LLC, Tiverton, RI 02878, USA; Michael_Ezrokhi@veroscience.com (M.E.); Yahong_Zhang@veroscience.com (Y.Z.); Shuqin_Luo@veroscience.com (S.L.)

**Keywords:** circadian, neuroendocrine, resetting, bromocriptine, dopamine, diabetes, insulin resistance, metabolic, vascular

## Abstract

The treatment of type 2 diabetes patients with bromocriptine-QR, a unique, quick release micronized formulation of bromocriptine, improves glycemic control and reduces adverse cardiovascular events. While the improvement of glycemic control is largely the result of improved postprandial hepatic glucose metabolism and insulin action, the mechanisms underlying the drug’s cardioprotective effects are less well defined. Bromocriptine is a sympatholytic dopamine agonist and reduces the elevated sympathetic tone, characteristic of metabolic syndrome and type 2 diabetes, which potentiates elevations of vascular oxidative/nitrosative stress, known to precipitate cardiovascular disease. Therefore, this study investigated the impact of bromocriptine treatment upon biomarkers of vascular oxidative/nitrosative stress (including the pro-oxidative/nitrosative stress enzymes of NADPH oxidase 4, inducible nitric oxide (iNOS), uncoupled endothelial nitric oxide synthase (eNOS), the pro-inflammatory/pro-oxidative marker GTP cyclohydrolase 1 (GTPCH 1), and the pro-vascular health enzyme, soluble guanylate cyclase (sGC) as well as the plasma level of thiobarbituric acid reactive substances (TBARS), a circulating marker of systemic oxidative stress), in hypertensive SHR rats held on a high fat diet to induce metabolic syndrome. Inasmuch as the central nervous system (CNS) dopaminergic activities both regulate and are regulated by CNS circadian pacemaker circuitry, this study also investigated the time-of-day-dependent effects of bromocriptine treatment (10 mg/kg/day at either 13 or 19 h after the onset of light (at the natural waking time or late during the activity period, respectively) among animals held on 14 h daily photoperiods for 16 days upon such vascular biomarkers of vascular redox state, several metabolic syndrome parameters, and mediobasal hypothalamic (MBH) mRNA expression levels of neuropeptides neuropeptide Y (NPY) and agouti-related protein (AgRP) which regulate the peripheral fuel metabolism and of mRNA expression of other MBH glial and neuronal cell genes that support such metabolism regulating neurons in this model system. Such bromocriptine treatment at ZT 13 improved (reduced) biomarkers of vascular oxidative/nitrosative stress including plasma TBARS level, aortic NADPH oxidase 4, iNOS and GTPCH 1 levels, and improved other markers of coupled eNOS function, including increased sGC protein level, relative to controls. However, bromocriptine treatment at ZT 19 produced no improvement in either coupled eNOS function or sGC protein level. Moreover, such ZT 13 bromocriptine treatment reduced several metabolic syndrome parameters including fasting insulin and leptin levels, as well as elevated systolic and diastolic blood pressure, insulin resistance, body fat store levels and liver fat content, however, such effects of ZT 19 bromocriptine treatment were largely absent versus control. Finally, ZT 13 bromocriptine treatment reduced MBH NPY and AgRP mRNA levels and mRNA levels of several MBH glial cell/neuronal genes that code for neuronal support/plasticity proteins (suggesting a shift in neuronal structure/function to a new metabolic control state) while ZT 19 treatment reduced only AgRP, not NPY, and was with very little effect on such MBH glial cell genes expression. These findings indicate that circadian-timed bromocriptine administration at the natural circadian peak of CNS dopaminergic activity (that is diminished in insulin resistant states), but not outside this daily time window when such CNS dopaminergic activity is naturally low, produces widespread improvements in biomarkers of vascular oxidative stress that are associated with the amelioration of metabolic syndrome and reductions in MBH neuropeptides and gene expressions known to facilitate metabolic syndrome. These results of such circadian-timed bromocriptine treatment upon vascular pathology provide potential mechanisms for the observed marked reductions in adverse cardiovascular events with circadian-timed bromocriptine-QR therapy (similarly timed to the onset of daily waking as in this study) of type 2 diabetes subjects and warrant further investigations into related mechanisms and the potential application of such intervention to prediabetes and metabolic syndrome patients as well.

## 1. Introduction

Circadian rhythms of central nervous system (CNS) dopaminergic activity have been implicated in modulating peripheral fuel metabolism via influences on (a) the hypothalamic regulation of the neuroendocrine axis targeting peripheral metabolic organs such as liver, adipose, endocrine pancreas, and muscle [1,2,3,4,5], and (b) striatal neurophysiology that manifests feeding behavior [6,7,8]. Typically, a diminution in the natural circadian peak in CNS dopaminergic activity facilitates the insulin resistant, obese state that is associated with cardiovascular disease, while the subsequent reinstatement of CNS circadian rhythm of dopaminergic activity reverses this condition [1,2,3,9,10,11].

Bromocriptine-QR is a unique quick release formulation of micronized bromocriptine, a potent dopamine D2 receptor agonist, which is FDA-approved to treat type 2 diabetes (T2D), indicated to be administered within 2 h of waking in the morning (the onset of daily locomotor activity and the natural peak of CNS dopaminergic activity) [12]. This circadian dopamine agonist therapy for type 2 diabetes provides only a brief morning pulse of dopamine agonist to the circulation, and yet reduces postprandial hyperglycemia across the three major meals of the day (i.e., up to 12 h after its administration) without raising the plasma insulin level [1]. The mechanisms operative in this response, as derived from preclinical studies, include (1) an amelioration of hypothalamic glucose sensing neurons’ loss of responsiveness to hyperglycemia required for post meal (insulin-stimulated) glucose disposal [3]; (2) a reduction in elevated sympathetic tone to reduce hepatic and peripheral insulin resistance [2,11,13]; (3) a reduction in leptin resistance [2,11,13], and (4) a simultaneous reduction in hypothalamic overactive neuropeptide Y and corticotropin releasing hormone (CRH) functions to improve peripheral glucose disposal [14]. Notably, this circadian anti-diabetes therapy has also been observed to rapidly (within 1 year) and markedly reduce (by 40–55%) adverse cardiovascular events in a large randomized trial of T2D subjects [15,16,17,18].

How these cardiovascular effects are manifested is not presently well understood. Inasmuch as circadian rhythms of neuroendocrine events, including autonomic balance, within the cardiovascular system play major roles in cardiovascular health including control of blood pressure, vascular endothelial function, cardiomyocyte biology, and daily cardiac rhythmicity [19,20,21,22,23,24,25], it was postulated that such neuroendocrine modulation by bromocriptine-QR may contribute to its observed beneficial effects on adverse cardiovascular outcomes. Since the impact of bromocriptine-QR on cardiovascular disease (CVD) was so rapid and increases or decreases in vascular oxidative and nitrosative stress can rapidly and significantly worsen or improve cardiovascular health, respectively, in individuals with dysmetabolism [26,27,28,29,30,31,32] (insulin resistance, obesity, T2D, or metabolic syndrome (a composite of obesity, dyslipidemia, hypertension, and associated low grade systemic inflammation)), this study investigated the potential impact of circadian-timed bromocriptine administration to improve vascular oxidative/nitrosative pathology in hypertensive SHR rats induced to generate metabolic syndrome by maintenance on a high fat diet. The SHR rat is well described to exhibit vascular reactive oxygen species/reactive nitrogen species (ROS/RNS) stress and arteriosclerosis [33,34,35,36,37].

Specifically, this study investigated the impact of such treatment upon aortic tissue (endothelium, smooth muscle cell) biomarkers of (a) vascular oxidative and nitrosative stress, including vascular NADPH oxidase 4 and inducible nitric oxide synthase (iNOS) protein levels, enzymes whose overactivation contributes to increase ROS/RNS [31,38,39,40,41,42,43,44,45,46,47] and (b) endothelial nitric oxide synthase (eNOS) uncoupling. Vascular eNOS uncoupling is a phenomenon of pathologically oxidative-induced altered eNOS structure resulting in the eNOS attenuation of nitric oxide (NO) production (the main cardioprotective product of eNOS that stimulates sGC) and simultaneous eNOS amplification of multiple ROS and subsequent RNS production [48,49,50,51,52], actions known to be potent inducers of rapid cardiovascular damage [26,27,28,29,30,31,32]. Such eNOS uncoupling is observed in hypertensive, insulin resistant, and T2D animals and humans, and is believed to represent the initial insult to the vasculature leading to CVD [26,27,28,29,30,31,32].

Secondly, in this animal model system, the time-of-day-dependent effects of bromocriptine (administered either at the daily peak of CNS dopaminergic activity in healthy animals on regular chow (Zeitgeber time (ZT) 13 h after light onset on daily 14 h photoperiods (which is diminished by high fat feeding)) or at the daily trough of CNS dopaminergic activity in healthy animals (ZT 19 h after light onset on daily 14 h photoperiods (which is abnormally elevated by high fat feeding))) on this vascular endpoint and additionally upon a) the composite of hyperinsulinemia, insulin resistance, liver lipid accretion, hypertension, hyperleptinemia, obesity, and plasma biomarker of increased systemic oxidative stress (the hallmarks of metabolic syndrome known to potentiate vascular pathology) and b) MBH mRNA expression of neuropeptide Y (NPY) and agouti related protein (AgRP), and associated glial cell genes that support such metabolism-regulating neurons, that potentiate metabolic syndrome [53,54,55,56,57], were investigated as well.

## 2. Results

### 2.1. Effect of ZT 13 versus ZT 19 Bromocriptine Treatment on Aortic Levels of Enzymes Regulating Tissue Redox Status

**Figure 1:**
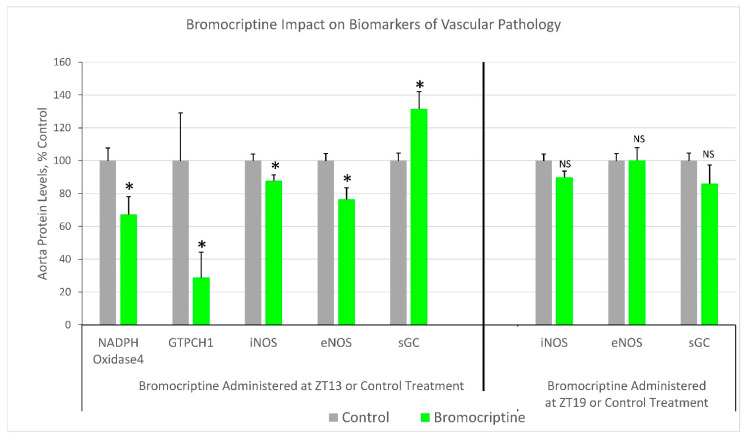
Impact of timed daily bromocriptine (at ZT 13 or ZT 19) or control treatment for 16 days on markers of vascular pathology. *—*p* < 0.05, 2-sided *t*-test; NS—not significant.

Bromocriptine treatment at ZT 13 reduced aorta tissue protein levels of the ROS/RNS generating enzymes iNOS (12%, *p* < 0.05) and NADPH oxidase 4 (33%, *p* < 0.03) relative to controls. Moreover, such ZT 13 bromocriptine treatment also reduced aortic protein level of the oxidative/nitrosative stress response protein, GTPCH 1 (71%, *p* < 0.05) versus control. Finally, ZT 13 bromocriptine treatment reduced aorta eNOS protein level (24%, *p* < 0.05) while simultaneously increasing aortic sGC protein level (32%, *p* < 0.02) versus control. Contrariwise, ZT 19 bromocriptine treatment did not significantly change aorta eNOS or iNOS protein levels and actually numerically reduced the sGC level, though not significantly. The assessment of NADPH oxidase 4 and GTPCH 1 were not obtained from the ZT 19 bromocriptine-treated animal samples due to the inadvertent loss of material for Western blot (Figure 1).

### 2.2. Effect of ZT 13 versus ZT 19 Bromocriptine Treatment on Cardiometabolic Parameters

**Figure 2:**
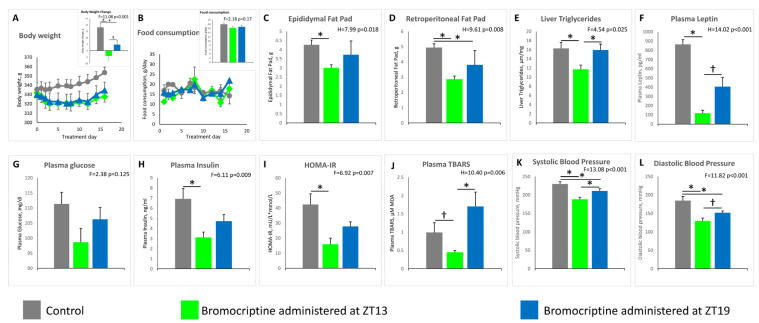
Impact of the timed daily bromocriptine (at ZT 13 or ZT 19) or control treatment for 16 days on metabolic syndrome parameters. Effects were examined using 1-way ANOVA (F) followed by pairwise post hoc (Holm–Sidak method) for normally distributed data, or Kruskal–Wallis (H) one-way analysis of variance on ranks test followed by Dunn’s pairwise multiple comparison procedures test for non-normal data, see Methods. Significant pairwise comparisons revealed from post hoc analysis or Dunn’s test are indicated by horizontal bars with *—*p* < 0.05, 2-sided; †—*p* < 0.05, 1-sided (see Results for details of individual parameter comparison *p* values).

Bromocriptine treatment at ZT 13 reduced plasma glucose by 11% (from 111 ± 4 to 99 ± 5 mg/dL, NS), and plasma insulin by 55% (from 6.9 ± 1.0 to 3.1 ± 0.5 ng/mL, *p* < 0.01) and resultantly reduced the HOMA-IR index by 62% (from 42 ± 7 to 16 ± 4 mU/L × mmol/L, *p* < 0.01) (treatment effect F = 2.38 *p* = 0.125; F = 6.11, *p* = 0.009; F = 6.92, *p* = 0.007, respectively), however, such treatment at ZT 19 was without significant effect on plasma glucose change (from 111 ± 4 to 106 ± 4 mg/dL, NS), plasma insulin change (from 6.9 ± 1.0 to 4.7 ± 0.7 ng/mL, NS), or HOMA-IR change (from 42 ± 7 to 28 ± 3 mU/L×mmol/L, NS). Similarly, bromocriptine treatment at ZT 13 reduced plasma TBARS by 55% (from 0.99 ± 0.26 to 0.45 ± 0.05 µM MDA, *p* < 0.05), (treatment effect H = 10.40 *p* = 0.006), yet was increased, but not significantly, when administered at ZT 19 (from 0.99 ± 0.26 to 1.70 ± 0.39 µM MDA). Resultantly, plasma TBARS values were 3.8-fold greater at bromocriptine at ZT 19 versus bromocriptine at ZT 13 (*p* < 0.01). Bromocriptine treatment at ZT 13 reduced plasma leptin by 86% (from 866 ± 50 to 117 ± 32 pg/mL *p* < 0.001), but not at ZT 19 (from 866 ± 50 to 406 ± 99 pg/mL, *p* =0.3) relative to controls (treatment effect H = 14.02 *p* < 0.001). The further 71% reduction in plasma leptin at ZT 13 versus ZT 19 was significant (*p* < 0.05). Bromocriptine treatment at ZT 13 reduced systolic BP (by 18%, from 230 ± 6 to 188 ± 6 mmHg, *p* < 0.001) as did such treatment at ZT 19 (by 9%, from 230 ± 6 to 211 ± 6 mmHg, *p* < 0.04) versus controls. The further 11% reduction in systolic blood pressure at ZT 13 versus ZT 19 was significant (*p* < 0.03) (treatment effect F = 13.08 *p* < 0.001). Bromocriptine treatment at ZT 13 reduced diastolic BP (by 30%, from 185 ± 11 to 130 ± 8 mmHg, *p* < 0.001) as did such treatment at ZT 19 (by 18%, from 185 ± 11 to 152 ± 5 mmHg, *p* < 0.02) versus controls. The further 15% reduction in diastolic blood pressure at ZT 13 versus ZT 19 was significant (*p* < 0.04) (treatment effect F = 11.82 *p* < 0.001) (Figure 2).

Bromocriptine treatment at ZT 13 reduced the epididymal fat pad by 29% (from 4.27 ± 0.26 to 3.01 ± 0.17 g, *p* < 0.03) and the retroperitoneal fat pad by 42% (from 4.94 ± 0.24 to 2.87 ± 0.20 g, *p* < 0.02), however, such treatment at ZT 19 did not significantly alter epididymal fat pad weight (from 4.27 ± 0.26 to 3.73 ± 0.75 g, *p* = 0.104) but did decrease the retroperitoneal fat pad weight (from 4.94 ± 0.24 to 3.81 ± 0.92 g, *p* < 0.05) versus control animals (treatment effect H = 7.99 *p* = 0.018 and H = 9.61 *p* = 0.008, respectively). Bromocriptine treatment at ZT 13 reduced the liver triglyceride (TG) content by 28% (from 16.3 ± 1.3 to 11.7 ± 0.9 μM/mg, *p* < 0.05), while such treatment at ZT 19 was without effect on liver TG (from 16.3 ± 1.3 to 15.9 ± 1.3 μM/mg) versus controls (treatment effect F = 4.54 *p* = 0.025). Control animals gained 18.1 g over the treatment period, while animals treated with bromocriptine at ZT 13 lost 4.3 g (*p* < 0.001 versus control), and animals treated with bromocriptine at ZT 19 gained 4.8 g (*p* < 0.03 versus control; *p* < 0.04 versus bromocriptine at ZT 13) (treatment effect F=11.08 *p* < 0.001). Food consumption was not different between the control, bromocriptine treatment at ZT 13, and bromocriptine treatment at ZT 19 groups (treatment effect F = 2.18 *p* = 0.17) (Figure 2).

### 2.3. Effect of ZT 13 versus ZT 19 Bromocriptine Treatment on MBH AgRP/NPY, Glial Cell Neuronal Support Factor, and Neuronal Plasticity Genes mRNA Levels

**Figure 3:**
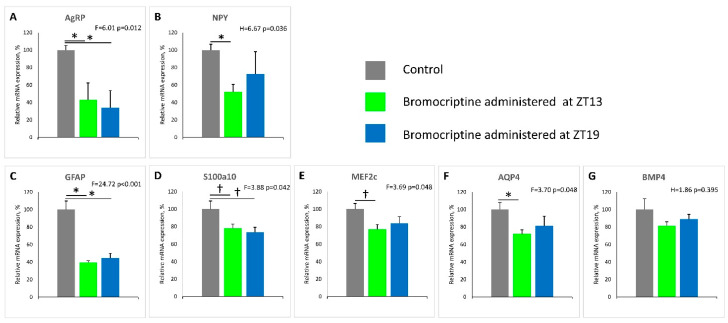
Impact of timed daily bromocriptine (at ZT 13 or ZT 19) or control treatment for 16 days on mediobasal hypothalamic (MBH) gene expression. Effects were examined using 1-way ANOVA (F) followed by pairwise post hoc (Holm–Sidak method) for normally distributed data, or Kruskal–Wallis (H) one-way analysis of variance on ranks test followed by Dunn’s pairwise multiple comparison procedures test for non-normal data, see Methods. The significant pairwise comparisons revealed from post hoc analysis or Dunn’s test are indicated by horizontal bars with *—*p* < 0.05, 2-sided; †— *p*< 0.05, 1-sided (see results for details of individual parameter comparison *p* values).

Bromocriptine treatment at ZT 13 reduced MBH mRNA levels of AgRP and NPY by 57% (*p* < 0.03) and 48% (*p* < 0.04), respectively, while such treatment at ZT 19 reduced the MBH AgRP level by 66% (*p* < 0.03) but was without significant effect on the NPY mRNA level versus control animals (treatment effect F = 6.01 *p* = 0.012 and H = 6.67 *p* = 0.036, respectively). Bromocriptine treatment at ZT 13 reduced MBH mRNA levels of Gfap, S100a10, Mef2c, and Aqp4 by 60% (*p* < 0.001), 22% (*p* < 0.04), 23% (*p* < 0.03), and 28% (*p* < 0.05), respectively, while only Gfap and S100a10 were significantly reduced by bromocriptine treatment at ZT 19 by 55%; (*p* < 0.001), and 26% (*p* < 0.04), respectively, relative to controls (treatment effect F = 24.72 *p* < 0.001; F = 3.88 *p* = 0.042; F = 3.69 *p* = 0.048; F = 3.70 *p* = 0.048, respectively) (Figure 3). MBH BMP4 mRNA levels were unaffected by bromocriptine treatment at either ZT 13 or ZT 19 (treatment effect H = 1.86 *p* = 0.395) (Figure 3).

## 3. Discussion

This study is the first to demonstrate that circadian-timed bromocriptine administration at the daily peak of CNS dopaminergic activity in healthy animals (ZT 13; that is diminished in insulin-resistant states [1,2,3]), can attenuate multiple in vivo enzymatic biomarkers of vascular tissue ROS and RNS stress, endothelial eNOS uncoupling, and total circulating oxidative stress (TBARS level), and such stress status is known to collectively precipitate CVD (Figure 1 and Figure 2). Moreover, such ZT 13 time-of-day-dependent bromocriptine effects upon reductions in vascular pathology were coupled to time-of-day-dependent bromocriptine reductions in (a) multiple pathophysiological components of metabolic syndrome including hyperinsulinemia, insulin resistance, hyperleptinemia, hypertension, increased liver TG content, and obesity, and (b) mediobasal hypothalamic NPY and AgRP mRNA expression, overactivations of which are known to induce metabolic syndrome [53,54,55,56]. By contradistinction, ZT 19 bromocriptine treatment, though producing reductions in body weight, retroperitoneal fat pad weight, and systolic and diastolic blood pressure (yet though none to the degree of ZT 13 bromocriptine treatment) did not significantly alter (improve) the levels of vascular eNOS, iNOS or sGC protein, plasma TBARS, epididymal fat store level, liver fat content, fasting plasma glucose, insulin or HOMA-IR (Figure 1 and Figure 2). Moreover, while ZT 13 bromocriptine treatment reduced both MBH AgRP and NPY mRNA levels, ZT 19 bromocriptine treatment only reduced MBH AgRP expression without effect on NPY mRNA (Figure 3).

**Figure 4:**
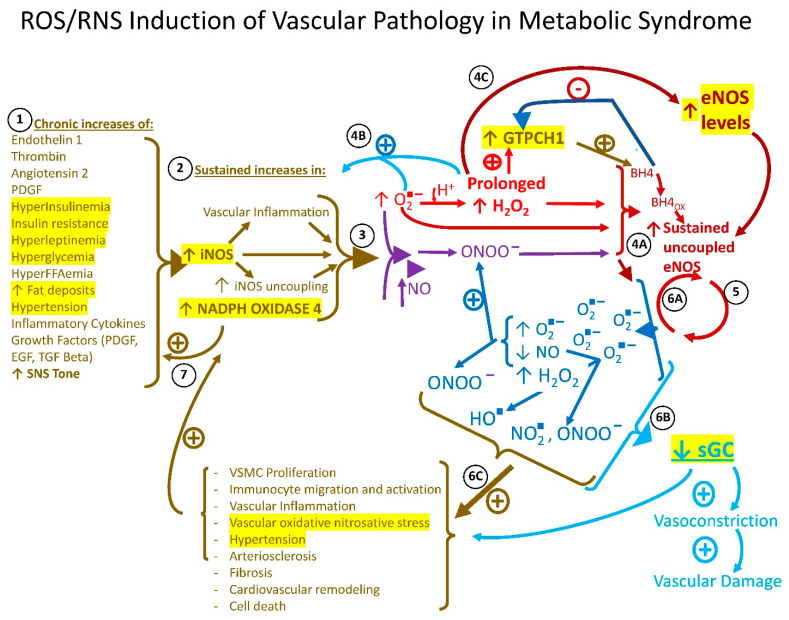
ROS/RNS induction of vascular pathology in metabolic syndrome. *Chronic* increases in several humoral, neural, and metabolic factors of metabolic syndrome listed in **1** potentiate sustained increases in the expressions of NADPH oxidase 4 and iNOS. **2**. Such increases in NADPH oxidase 4 and iNOS activities produce increases in oxygen free radical (O_2_^−^) that can react with H^+^ to form hydrogen peroxide (H_2_O_2_) and with nitric oxide (NO) to form peroxynitrite (ONOO^−^). **3**. All three of O_2_^−^, H_2_O_2_, and ONOO^−^ lead to eNOS uncoupling via oxidation of tetrahydrobiopterin (BH_4_ to BH_4ox_ (BH_2_)) and several other mechanisms. **4A**. These ROS/RNS can also feedback to indirectly potentiate further increases in NADPH oxidase 4 and iNOS. **4B**. *Prolonged* increases in H_2_O_2_ stimulate an increase in eNOS expression that ultimately becomes uncoupled under the concurrent environment of increased ROS/RNS. **4C**. The increased levels of uncoupled eNOS decrease its NO production and generate increased O_2_^−^ levels that again react with H^+^ and NO to form H_2_O_2_, ONOO^−^, hydroxyl radical (OH^−^), nitrogen dioxide (NO_2_^−^) and several other ROS/RNS species. **5**. These uncoupled eNOS-generated ROS/RNS feedback to uncouple more eNOS; **6A**, and feedforward (in conjunction with decreased NO) to decrease the expression and activity of soluble guanylate cyclase (sGC), resulting in a decreased effect of the sGC product, cGMP, to promote vascular tissue vasodilation and health. **6B**. These uncoupled eNOS-generated ROS/RNS products, along with those formed via increased NADPH oxidase 4 and iNOS feedforward to also directly cause damage to the vasculature (endothelium and smooth muscle cells) as listed in **6C**. **7**. Inflammatory cytokines and growth factors from infiltrating immunocytes and proliferating vascular smooth muscle cells further stimulate NADPH oxidase 4 and iNOS and these enzymes also feedback to stimulate these same actions. The metabolic syndrome milieu initiates and maintains the vascular production of ROS/RNS via multiple internal positive feedback and feedforward lops that ultimately lead to vascular damage and CVD. Prolonged increases in H_2_O_2_ production from NADPH oxidase 4, iNOS, and uncoupled eNOS stimulate the increased expression of GTPCH 1, the rate-limiting enzyme in BH_4_ synthesis, and BH_4_ is itself an end product, negative feedback inhibitor of GTPCH 1. As BH_4_ becomes oxidized by the prevailing ROS/RNS stress, less of it is available to inhibit GTPCH 1. Increased levels of GTPCH 1 are a biomarker of tissue pro-oxidative/nitrosative and proinflammatory state. NO_2_^−^—nitrogen dioxide; OH^−^—hydroxyl radical; PDGF—platelet-derived growth factor; EGF—epidermal growth factor; TGFβ—transforming growth factor beta; SNS—sympathetic nervous system; VSMC—vascular smooth muscle cell; BH_4_—tetrahydrobiopterin; BH_4 ox_—oxidized tetrahydrobiopterin; 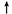
—increases; 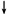
—decreases; 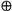
—stimulates; 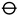
—inhibits; 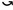, 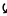, 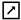, 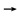, 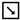, 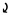, 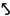, 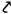, 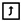, 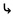 leads to; 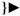, 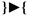 collectively leads to these collective events. Enzyme levels and cardiometabolic parameters measured in the study are highlighted in yellow.

The pathophysiology of chronic vascular tissue ROS/RNS stress encompasses a viscous positive feedback loop among several enzymes that work in concert to continually amplify vascular tissue ROS and RNS leading to CVD [26,27,28,29,30,31,32,38,39,40,41,42,43,44,45,46,47,48,49,50,51,52]. As it relates to the specific investigations of this study, this pathophysiology may be summarized as follows. Under normal healthy circumstances, vascular tissue redox state exists as a balance between moderate prooxidative species activities that promote normal cell functions such as growth, repair, and energy production and host defense against pathogens on the one hand via a low level of oxidative species (oxygen free radical (O_2_^−^), hydrogen peroxide (H_2_O_2_), peroxynitrite (ONOO^−^) and several others) and anti-oxidative activities that prevent a chronic over-amplification of such prooxidative activities that are damaging to the vasculature on the other hand [58,59]. Vascular (endothelial and smooth muscle cell) NADPH oxidase 4 generates low levels of ROS that normally function to stimulate numerous transcription factors involved in gene expressions that are critical in the normal regulation of cellular biology. However, under chronic stimulation from a metabolic syndrome milieu including obesity, hyperglycemia, hyperinsulinemia, hyperFFAemia, hypertension, increased circulating levels of growth factors such as transforming growth factor β, platelet-derived growth factor, and epidermal growth factor, and increased circulating and local vascular immunocyte inflammatory cytokines such as tumor necrosis factor α, IL1, and IL6 and increased thrombin and angiotensin II, all which increase NADPH oxidase 4 expression, the enzyme generates sustained high levels of ROS O_2_^−^ and subsequent H_2_O_2_ [31,38,39,40,41,58,60]. The NADPH oxidase 4-derived O_2_^−^ can also react with NO to generate ONOO^−^ via O_2_^−^ reaction with immediate vicinity NO (which is derived from overexpressed iNOS activity) which, along with sustained high levels of O_2_^−^ and H_2_O_2_, collectively damage the vasculature [26,27,28,29,30,31,32,38,39,40,41,42,43,44,45,46,47,48,49,50,51,52,60,61,62] (see below). Similarly, vascular iNOS, a smooth muscle cell and immunocyte enzyme that exists as a homodimer that converts arginine and oxygen to citrulline and NO, normally produces acute moderate amounts of NO and ROS when induced by cytokines or bacteria to facilitate normal smooth muscle cell function (including vasodilation) and immunocyte bactericidal activity, respectively. However, the chronic stimulation of iNOS by the metabolic syndrome milieu leads to markedly increased levels of NO and O_2_^−^ which react to form ONOO^−^, a potent prooxidant, which along with O_2_^−^ and H_2_O_2_, uncouples the iNOS dimer to alter its functional activity to inappropriately generate even higher levels of O_2_^−^ [41,42,43,44,45,46,47]. Under situations of chronically elevated NADPH oxidase 4 and iNOS levels and activities, the increased generation of their ROS and RNS products, particularly H_2_O_2_ and peroxynitirite, at high levels can function to directly and indirectly (via growth factor stimulation) feedback to stimulate their own and each other’s further enzyme expression and subsequent further ROS/RNS generating action [60,61,62,63,64,65]. Additionally, the chronic elevation/activation of these ROS/RNS products from these two ROS/RNS generating enzymes feedforward to alter endothelial eNOS activity to both increase its expression level and to uncouple the enzyme (as with iNOS) and subsequently increase ROS production (see below) [60,61,62,66,67,68,69]. Moreover, these iNOS and NADPH oxidase 4 ROS/RNS products can directly inhibit the expression and activity of sGC, the vasodilatory and vascular health-promoting enzyme of the vasculature that is stimulated by eNOS-derived NO. Vascular eNOS under the influence of the cofactor tetrahydrobiopterin (BH_4_), exists as a homodimer that converts arginine and oxygen to citrulline and NO. At constitutively low levels, eNOS-derived NO acts (in part via the stimulation of sGC) to inhibit several local inflammatory processes, vascular smooth cell proliferation, excessive adhesion molecule aggregation, apoptosis, and arterial stiffness, to stimulate endothelial cell health and vessel relaxation [59,61]. However, under the chronic condition of the metabolic syndrome milieu described above, with its incident overexpression of vascular tissue iNOS and NADPH oxidase 4 that act to sustain increases in local ROS and RNS, the eNOS dimer also becomes uncoupled in part due to the ROS/RNS oxidation of BH_4_ (to BH_2_), changing eNOS enzymatic activity to attenuate NO and amplify O_2_^−^ production. This subsequent increased O_2_^−^ production again facilitates increased H_2_O_2_ and RNS (peroxynitrite and nitrogen dioxide, among others) generation that all induce the further expression and subsequent uncoupling of eNOS [48,49,50,51,52,60,61,62,63] and the positive feedback loops become amplified with each cycle (Figure 4). Resultantly, the cardioprotective effects of the low-level activity of these enzymes (NADPH oxidase 4, iNOS, and eNOS) are converted by the metabolic syndrome milieu factors to a cardiovascular damaging status via an enzymatic interplay of self-sustaining high-level activity.

In this vascular redox system, the GTPCH 1 enzyme, a well-established protein biomarker of vascular tissue inflammation [43], functions as the rate-limiting enzyme for BH_4_ synthesis and is induced/activated by both elevated H_2_O_2_ and reduced BH_4_ levels under ROS/RNS stress conditions (that oxidize BH_4_ to BH_2_ which in turn facilitates eNOS uncoupling and the subsequent production of H_2_O_2_) [70]. However, under such ROS/RNS stress, GTPCH1 simply cannot keep up with the demand for BH_4_ and merely makes the ROS/RNS stress worse by supplying more BH_4_ for it to only to be oxidized and thus feedforward to uncouple eNOS, in turn generating more ROS and RNS to continue the cycle via the stimulation of iNOS and NADPH oxidase and the uncoupling of iNOS and eNOS [43]. Consequently, under the metabolic syndrome milieu, vascular tissue elevations of NADPH oxidase 4 and iNOS and uncoupled eNOS collude to chronically produce high levels of several ROS and RNS species including O_2_^−^, H_2_O_2_, ONOO^−^, and its reaction products OH^−^ (hydroxyl radical), CO_3_^−^ (carbonate radical), and NO_2_, (nitrogen dioxide) among several other ROS and RNS intermediates throughout the vasculature.

At the cellular level, the chronic elevation of such ROS/RNS throughout the vasculature tissues alter cellular signaling pathways, enzyme activities, mitochondrial function, and DNA integrity ultimately leading to increased cellular necrosis and apoptosis, adverse extracellular remodeling, and fibrosis while further promoting inflammatory immune cytokine responses, to stimulate the upregulation of the very enzymes responsible for ROS/RNS generation [71,72,73,74,75]. Such cellular consequences of sustained increases in vascular tissue ROS/RNS can potentiate a range of vascular tissue pathologies including inflammation, endothelial dysfunction, smooth muscle cell proliferation, immunocyte migration and activation, extracellular matrix deposition, fibrosis, angiogenesis, cardiovascular remodeling, hypertension, atherosclerosis, arteriosclerosis, myocardial ischemia reperfusion injury, and cardia failure [26,27,28,29,30,31,32,76,77,78,79,80,81,82,83]. If left unabated, sustained increases in vascular tissue ROS/RNS can destroy the cardiovascular system.

Bromocriptine administered at ZT 13 reduced aortic protein levels of iNOS and NADPH oxidase 4, two enzymes whose overexpression is well known to generate vascular ROS/RNS stress and be associated with hypertensive, insulin-resistant states as described above. Such treatment also reduced GTPCH1, an enzyme amplified in response to existing local ROS/RNS stress (particularly elevated H_2_O_2_) and reduced by high BH_4_ levels (feedback inhibition) suggesting a reduction in local tissue ROS/RNS stress by ZT 13 bromocriptine treatment. Coincidentally, such treatment reduced the protein level of eNOS and increased the protein level of soluble guanylate cyclase (stimulated by NO generated by eNOS), a tell-tail sign of reversal of eNOS uncoupling [38,49,50,51,52,69], particularly when concurrent with the GTPCH1 protein level reduction and supported by decreases in iNOS and NADPH oxidase protein levels. That is, only a reduction in the uncoupled pro-oxidative form of the eNOS protein level would be expected to generate an increase in the sGC level since eNOS-derived NO stimulates and O_2_^−^ inhibits sGC expression. This is particularly the case in the presence of reduced amounts of the two major prooxidant enzymes, iNOS and NADPH oxidase 4, which reduction would both reduce eNOS uncoupling, decrease eNOS expression (via relief from H_2_O_2_ overstimulation) and increase sGC expression (in response to increased eNOS-derived NO and normalized (decreased) H_2_O_2_ and other ROS/RNS levels as described above). In T2D, hypertensive, and atherosclerotic humans that often suffer from vascular ROS/RNS stress, the levels of endothelial eNOS are actually elevated due to elevated H_2_O_2_ overstimulation of its expression, but the enzyme is largely uncoupled, generating damaging ROS/RNS as described above [38,49,50,51,52,69]. The marked GTPCH 1 level reduction following ZT 13 bromocriptine treatment, signifying a reduced pro-inflammatory/pro-oxidative state, is consistent with all these enzyme protein level changes and thus with the conclusion that ZT 13 bromocriptine treatment reduces excessive ROS/RNS stress on the vasculature. Finally, consistent with a ZT 13 BC reduction in vascular oxidative stress is the observation of a >50% reduction in plasma TBARS level, a circulating biomarker of systemic oxidative stress. Contrariwise, these beneficial effects of bromocriptine on aortic eNOS and sGC protein levels and plasma TBARS levels were absent, and potentially worsened, when administered at ZT 19.

What factors may be involved in this circadian-timed bromocriptine reduction in vascular ROS/RNS stress? In this regard, as described above, it is well recognized that the obese, hypertensive, hyperinsulinemic-insulin-resistant state (metabolic syndrome) is a major risk factor for vascular ROS/RNS stress and CVD. Vascular endothelial and smooth muscle cell levels of both NADPH oxidase 4 and iNOS are amplified by the hyperinsulinemic-insulin-resistant, hypertensive state characteristic of SHR rats on a high fat diet as shown in this study [33,34,35,36,37,84]. Moreover, each of elevated sympathetic tone, hypothalamic-pituitary axis (HPA) overactivity, and leptin resistance characteristic of metabolic syndrome, particularly in SHR rats, directly and indirectly potentiate the metabolic syndrome, CVD, and vascular ROS/RNS stress, by inducing hypertension, immunocyte inflammatory cytokine secretion, fueling adipose, and liver inflammatory cytokine production and secretion, and stimulating free fatty acid mobilization to increase fatty liver, insulin resistance, and vascular inflammation (reviewed in [13]). Such activities in turn stimulate vascular iNOS and NADPH oxidase 4, thus generating ROS/RNS that lead to more vascular inflammation, cellular damage, and matrix remodeling, and ultimately vascular disease as outlined above. Among these pathological neuroendocrine factors, elevated sympathetic tone stands out as a singular pathology that can itself potentiate the entire metabolic syndrome and vascular disease thereof (reviewed in [12]). Bromocriptine administration at ZT 13 has previously been demonstrated to ameliorate the obese, hyperinsulinemic-insulin-resistant state with concurrent reductions in elevated sympathetic tone, hypertension, liver inflammatory cytokine transcription factor production, liver fat content, and hyperleptinemia in SHR rats on regular chow [13]. This study extends those findings to demonstrate that such ameliorative effects of ZT 13 bromocriptine treatment are also demonstrable in this animal model, even while held on a high fat diet. Importantly, the ameliorative actions of ZT 13 bromocriptine upon metabolic syndrome were substantially reduced or absent when administered at ZT 19. Previous studies indicate that this effect of ZT 13 bromocriptine treatment on metabolic syndrome occurs via the normalization of overactive norepinephrine (NE) and serotonin (5HT) activities at the ventromedial hypothalamus (VMH) and of overactive NPY and CRH activities at the paraventricular nucleus (PVN) to reduce elevated sympathetic outflow to the periphery (reviewed in [1,2,85]), as well as to normalize VMH glucose sensing to improve glucose tolerance and insulin resistance [3]. We therefore investigated the possible impact of ZT 13 versus ZT 19 bromocriptine upon MBH AgRP and NPY expressions since hypothalamic arcuate nucleus NPY and AgRP expressions are upregulated during the fattening phase in response to a high fat diet [55,86,87] or to the initiation of the seasonal obese condition [88], and such neuronal overactivation markedly stimulates the hyperinsulinemic-insulin-resistant obese condition [53,54,55,56]. Moreover, previous studies have demonstrated that dopamine agonist treatment can reduce hypothalamic NPY levels in the ob/ob leptin-deficient genetic model of metabolic syndrome [14].

Among ZT 13 bromocriptine-treated animals, the hypothalamic levels of AgRP and NPY mRNAs were markedly reduced by 57 and 48%, respectively, relative to controls, and such reductions were concurrent with a marked 86% reduction in circulating leptin levels. Inasmuch as leptin and insulin are potent inhibitors of arcuate neuronal AgRP and NPY expression and activity under normal metabolic conditions [89,90,91,92], and these neurons lose their responsiveness to such leptin and insulin actions during high fat feeding and obesity [93,94,95,96,97], these present findings strongly imply an improvement in AgRP/NPY neuronal leptin and insulin sensitivity to reduce AgRP and NPY expression, even during prolonged high fat diet feeding by ZT 13 bromocriptine treatment. However, although ZT 19 bromocriptine treatment similarly reduced MBH AgRP expression as ZT 13 BC, it was without effect upon MBH NPY mRNA expression and plasma insulin and leptin, (see Figure 2 and Figure 3). These marked ZT 13 bromocriptine treatment effects to reduce elevated hypothalamic AgRP and NPY mRNA levels and plasma leptin and insulin are consistent with and can provide for such time-of-day effects on both overall dysmetabolism and biomarkers of vascular pathology in this study.

Changes in CNS (hypothalamic) glial cell and neuronal function, morphology, and anatomy (termed neuronal plasticity) that provide for marked seasonal changes in CNS and neuroendocrine actions and functions at different times of the year to drive seasonal physiology such as seasonal changes in reproduction, behavior, immunity, and metabolism, have been suggested to be driven in part by CNS circadian dopaminergic–pacemaker clock interactions [85]. Additionally, adverse neuronal plasticity changes induced by high fat diet are associated with both reduced brain dopaminergic function [8,98,99] and altered AgRP/NPY neuronal activity [100]. We therefore investigated, as an initial exploration, the possible influence of ZT 13 versus ZT 19 bromocriptine treatment upon MBH mRNA expression of Gfap, S100a10, Bmp4, and Aqp4 genes that code for proteins central to glial cell–neuronal metabolism/growth and protection functions (reviewed in [101]) and Mef2c, a gene product that influences neuronal plasticity [102] as a window into time-of-day dopamine agonist influence on hypothalamic neuronal plasticity mechanisms. Previous notable studies by M.A. Bentsen et al. [101] have demonstrated that the long-term anti-diabetic effect of fibroblast growth factor 1 (FGF 1) involves the short-term (1–5 day) induction followed by the longer term (14–42 day) attenuation of these genes in the MBH area of treated diabetes mice. In this study as well, in fact, the expression levels of Mef2c, Gfap, S100a10, and Aqp4 were all reduced after 14 days of ZT 13 bromocriptine treatment, while only Gfap and S100a10 were reduced by ZT 19 bromocriptine treatment (Figure 3). These findings suggest that circadian-timed bromocriptine administration may influence MBH neuronal plasticity in a manner that associates with ameliorated overexpression of AgRP and NPY to improve metabolism in high fat diet-induced metabolic syndrome. Speculatively, it may be that such ZT 13 bromocriptine treatment induced these genes earlier in the course of therapy as does FGF1, with subsequent attenuation once such neuronal morphology/function has been shifted, though much more work is needed to delineate the details of this association.

It must be recognized that this study design investigated time-of-day-dependent influences of dopamine agonist therapy upon vascular biology, metabolism, and hypothalamic neuroendocrine status, but did not investigate the influences of such time-of-day administrations upon either the circadian systems that may regulate these responses (e.g., suprachiasmatic nucleus circadian output to the neuroendocrine axis) or circadian expressions of the responses themselves (e.g., circadian rhythm of vascular tissue ROS/RNS enzyme activity, liver or adipose, metabolism blood pressure, hypothalamic NPY level). Now, having these present data in hand, it would be prudent to investigate the CNS and peripheral circadian systems targeted by this therapy. To do so, studies of the effects of ZT 13 bromocriptine treatment upon circadian expressions of clock and clock-regulated metabolic and immune genes in both the CNS pacemaker system and neuroendocrine target tissues of the vasculature, liver, adipose, and muscle need to be conducted. However, the existing fund of information on the dopamine regulation of circadian rhythms can offer some critical insights into how the present study time-of-day-dependent effects of bromocriptine to improve vascular pathology and dysmetabolism may be manifested, and given the marked CVD event rate reduction observed in clinical studies of bromocriptine-QR, are necessarily reviewed herein as a digression into dopamine–clock interactions as follows.

Among vertebrates, the organization of the CNS control of whole-body metabolism is indispensable for adaptation to changing environmental conditions of energy supply to maximize survival [103,104,105,106,107,108]. This is best exemplified among animals in the wild under natural conditions where such CNS neurophysiology evolved. Vertebrates across the planet (within and outside of the temperate zones of the world) manifest annual cycles of metabolism oscillating between the lean and obese states when food availability is plentiful and scarce, respectively. Animals anticipate the low food availability season by shifting to a lipid accretion anabolic yet insulin-resistant state in preparation. The obese insulin-resistant condition allows for the maintenance of a hepatic glucose supply to the CNS when little to zero is available in the environment and the hyperinsulinemic-lipogenic/obese condition supports the energy demands of the peripheral tissues (e.g., muscle) via free fatty acid mobilization, while insulin resistance shunts hepatic glucose supply to the CNS [108]. The seasonal shift to and from the obese, insulin-resistant state can still be observed under laboratory conditions of constant food supply/consumption and photoperiod throughout the year (same hours of light within the day across seasons). Vertebrates possess a timing mechanism to drive peripheral fuel metabolism (anabolism) either towards lipid accretion or protein turnover during the obese and lean seasons, respectively. Studies of such vertebrates, from teleosts to mammals, have identified critical roles for a dopamine-regulated CNS circadian pacemaker circuit (including the suprachiasmatic nucleus (SCN)) and cellular circadian rhythms in the expression of seasonal changes in physiology, including metabolism (all reviewed in [108]).

In this regard, seminal work in circadian rhythms’ regulation of vertebrate physiology by A.H. Meier and colleagues demonstrated that across representative species of all the major vertebrate classes, mimicking the circadian phase relation of peak plasma prolactin and corticosteroid hormone levels of a particular season, by injection of these hormones at these times of day, produced that seasonal metabolic condition irrespective of the actual time of year which effect persists long after (e.g., months) the termination of a short (~10 day) treatment ([108,109,110,111,112]). Importantly, such seasonal changes in plasma prolactin and corticosteroid hormone levels, observed among species of several vertebrate classes and demonstrated to regulate fuel metabolism as a function of their temporal organization, have been documented in obese versus lean humans as well [113].

The impact of this circadian clock system on metabolism in seasonal animals [108,109,110,111,112,113,114,115] was also demonstrable upon the metabolic changes associated with aging, as demonstrated in aged male Sprague Dawley rats. Injections of prolactin and corticosteroid into older obese, insulin-resistant rats at times of day they naturally peak in the blood of younger insulin-sensitive rats reversed the aging-associated obesity and insulin resistance, an effect which persisted for 11–14 weeks after the termination of treatment [116]. Such profound and long-lasting effects on whole-body physiology suggested that the prolactin and corticosteroid injections were not solely acting directly on multiple tissues of the body but were resetting a central clock pacemaker mechanism for the control of chronobiology throughout the animal via multiple circadian expressions (neuroendocrine stimulus (e.g., hormone or neurotransmitter) and response (hormone and/or neurotransmitter receptor) rhythm interactions), including the circadian rhythms of plasma prolactin and corticosteroid hormone levels [115,117,118]

Inasmuch as prolactin activates the rate-limiting enzyme in dopamine synthesis (tyrosine hydroxylase) [119] and corticosteroid activates the rate-limiting enzyme in serotonin synthesis (tryptophan hydroxylase) [120], it was postulated that the circadian-timed injections of prolactin and corticosteroid to reset annual metabolism (and physiology) were operating to set the phases of CNS dopaminergic and serotonergic neuronal activities that govern chronobiological output to the body via the neuroendocrine axis [115,121]. This postulate was affirmed by studies, reproduced among species of several vertebrate classes, demonstrating that the seasonal metabolic effects of specific circadian-timed injections of prolactin and corticosteroid hormone could be replaced/recapitulated by such similarly timed injections of l-DOPA (the precursor for dopamine) and 5-hydroxytryptophan (the precursor for serotonin), respectively [118,122,123,124]. Moreover, the circadian rhythms of dopamine and serotonin activities at the SCN, the seat of the biological pacemaker system within the CNS for chronobiological organization within the body, differ between seasonally obese, insulin-resistant and seasonally lean, insulin-sensitive mammals [9].

Inasmuch as the circadian peaks of dopaminergic input and activity at the SCN are markedly diminished in seasonal and diet-induced models of the obese, insulin-resistant state [2,9], we explored the cardiometabolic effects of circadian-timed dopamine administration directly to the SCN in obese, insulin-resistant hypertensive SHR rats held on a high fat diet. Such animals were treated with SCN-directed dopamine treatment either at the time of day it peaks in the SCN of healthy, lean, insulin-sensitive animals (one hour before light offset and the initiation of daily waking and locomotor activity, ZT 13) or several hours after this time period, during its natural circadian trough level and activity at the SCN (ZT 19) [2]. It was found that the daily administration of dopamine directly to the SCN of such animals for 14 days reversed the cardiometabolic syndrome of the animals held on high fat diet; however, the same treatment administered outside this daily peak interval but during the daily trough of such dopaminergic activity was completely ineffectual in this regard. Moreover, such appropriately timed administration of bromocriptine, a potent sympatholytic dopamine D2 receptor agonist, reverses the diabetogenic defect in hypothalamic glucose sensing induced by a high fat diet when it was administered within the window of the natural daily peak in SCN area dopaminergic activity (ZT 13) but not outside of it [3]. These findings are consistent with a long series of preclinical and clinical studies demonstrating that high fat feeding induces reductions in brain dopaminergic activity [7,98,99,125,126,127]. CNS circadian dopaminergic function appears to be atop a hierarchical system to regulate the circadian control of physiology, including metabolism that other signals (e.g., prolactin) either interact with or are to an extent subservient to. In keeping with such a postulate, we observed the bromocriptine reversal of metabolic syndrome in animal models of metabolic syndrome having normal to low plasma prolactin levels such as ob/ob and db/db mice [128,129], and even in animals with diminished α-melanocyte stimulating hormone (αMSH)–melanocortin-4 receptor activation [130], as have others more recently [131]. This is not unexpected, as these disrupted prolactin (and MSH) clock signals feed into a clock system to potentiate the dysfunction of circadian dopaminergic activity as a function of their altered circadian expressions [85,108].

If the loss or diminution of the daily peak in CNS dopaminergic activity rhythm is a primary signal to the clock circuit to drive its output control of metabolism towards metabolic syndrome, it is not surprising that a loss of singular signals (e.g., prolactin) in a redundant and subservient hierarchical organization of input signaling to the clock pacemaker circuit that are upstream from the dopaminergic input would be inconsequential to direct dopaminergic receptor replacement therapy for the metabolic syndrome. All said, it must also be appreciated that the presence of hyperprolactinemia is well documented to be associated with and potentiate metabolic syndrome and cardiovascular disease in animals and humans [132,133,134,135,136,137,138], which importantly, can be manifested via chronic prolactin inhibition of brain dopaminergic activity [139]. As such, the correction of abnormal elevations in plasma prolactin, where it exists in metabolic syndrome patients, and the reestablishment of its normal circadian variation, is a metabolic benefit of circadian bromocriptine therapy [135,138,140]. Finally, inasmuch as the circadian rhythm of plasma prolactin has marked influences upon immune function [141], and bromocriptine is a strong inhibitor of pituitary prolactin secretion, the appropriate circadian administration time of bromocriptine to treat metabolic disease must also take into account the functions of circadian plasma prolactin upon immunity (and several of its other functions—e.g., in reproductive physiology) so as not to disrupt them. Consequently, in total, the available evidence suggests that ZT 13 bromocriptine treatment resets the circadian peak of CNS dopaminergic activity of metabolic syndrome animals back to normal to normalizes the clock circadian pacemaker output in a manner that resets the circadian organization of the neuroendocrine axis controlling metabolism towards the normal non-metabolic syndrome state, while such drug therapy at ZT 19 simply does not because it functions to actually *prevent or disrupt* the normal central circadian organization governing normal peripheral metabolism (producing a gradation of lesser (or adverse) effects versus ZT 13 treatment) (analogous to sleep/wake disruption) (reviewed in [1]).

The current effects of bromocriptine administration to metabolic syndrome SHR rats at a daily time prior to or near the daily peak of CNS dopaminergic activity in healthy animals (onset of darkness; ZT 13 in animals held on a 14 h daily photoperiod) to reverse metabolic syndrome are similar to those observed among high fat fed mice wherein the effects of bromocriptine treatment to improve both glucose tolerance and obesity were better at ZT 11 (animals held on a 12 h daily photoperiod) than when administered at other times of day [131]. Interestingly, the study also demonstrated the effectiveness of ZT 11 bromocriptine to improve glucose tolerance (but not obesity) in clock gene knockout mice held on a high fat diet, leading the authors to conclude that circadian response systems were not required in the metabolic response to bromocriptine. However, it should be realized that clock-less mice still exhibit circadian rhythmicity at both the whole animal and SCN levels [142,143,144] due to the expression of Npas2, a clock homolog that can act as clock in many respects in its absence. However, these findings are worthy of further investigation. Most importantly, as these mouse data relate to the present study’s findings and the related clinical results that bromocriptine-QR reduces hyperglycemia, insulin resistance, and adverse cardiovascular outcomes in T2DM subjects when administered within 2 h of waking, it is particularly noteworthy that the effect of bromocriptine to improve insulin resistance in obese individuals was present when administered at the onset of waking (time of day of natural peak CNS dopaminergic activity in healthy individuals (analogous to ZT 13 bromocriptine treatment in nocturnal animals of the present study)), but not when administered late in the day [145].

Moreover, in this regard, it is relevant that pharmacological (alpha-methyl-para-tyrosine) reduction in brain dopamine levels for only a day or two to reduce the early morning hours CNS dopamine levels in young healthy individuals precipitates insulin resistance after only a single day or two of such treatment [146,147]. Finally, it should be noted that low brain dopaminergic activity has been associated with (a) homozygosity or heterozygosity for the Taq1 allele of the dopamine D2 receptor, which renders it less functional [148]; (b) disruptions to the sleep–wake cycle [149]; and (c) psychosocial stress [150] that each are associated with weight gain, glucose intolerance, and/or insulin resistance [148,151,152,153,154]. The present study findings suggest that the low brain dopaminergic activity associated with sleep–wake disorders and psychosocial stress may contribute to the risk of vascular pathology that these disorders carry.

## 4. Conclusions

In conclusion, circadian-timed ZT 13 bromocriptine administration to hypertensive, metabolic syndrome SHR rats reduced biomarkers of aortic ROS/RNS stress and circulating oxidative stress coupled to the reversal of the metabolic syndrome and reductions in MBH NPY and AgRP mRNA expressions, as well as influenced several MBH genes associated with neuronal plasticity, while ZT 19 bromocriptine administration was largely without effect on the vast majority of these test parameters.

Given that vascular ROS/RNS stress is a major contributor to cardiovascular disease, and that circadian-timed bromocriptine-QR therapy (timed to onset of daily activity as in this study) produced marked reductions in cardiovascular adverse events in a large randomized clinical trial of T2D subjects, the current study findings suggest that the influence of circadian-timed bromocriptine upon vascular ROS/RNS may contribute to such clinical results with this therapy. The present findings also suggest that improvement in CVD outcomes among pre-diabetes, metabolic syndrome patients may be observed with this therapeutic approach.

## 5. Materials and Methods

### 5.1. Animals

Eight-week-old male spontaneously hypertensive rats (SHR) were purchased from Taconic Biosciences (Hudson, NY, USA), and were housed in our climate-controlled animal care facility and maintained on 14 h daily photoperiods (14 h light/10 h dark) starting at 8 weeks of age and maintained under such photoperiod for the entire study. At 10 weeks of age, rats were switched from standard rodent chow to high fat diet (HFD) (Research Diets, New Brunswick, NJ, USA rodent diet Catalog # D12492 with 60% kcal from fat, 20% kcal from protein, 20% kcal from carbohydrate) and drinking water ad libitum throughout the entire study period. At the initiation of drug administration, animals were 16 weeks of age. Male SHR rats of this strain and age are severely hypertensive [13,57].

### 5.2. Experimental Design

Following 6 weeks of high fat diet feeding, 16 week-old SHR rats were randomized to one of three treatment groups: (1) bromocriptine (10 mg/kg/day) (70/30 water:ethanol by volume) (N = 8) administered intraperitoneally at 13 h after light onset (Zeitgeber time (ZT) 13) (a time of day of peak dopamine release at the area of the SCN clock in nocturnal rodents [10]); (2) bromocriptine, similarly prepared and administered as those at ZT 13 but given to animals at ZT 19 (N = 8) (a time of day of trough dopamine release at the SCN area in nocturnal rodents) under non-circadian disrupting red light (so as not to provide a white light pulse to the animals during the dark phase of the daily light/dark cycle); or (3) control treatment of similar measures/interactions at each time of day (N = 8), daily for 16 days. Food consumption and body weight were recorded over the 16-day treatment period. Measurements of blood pressure were taken at 4 h after light onset on the day before treatment initiation and again 14 days after treatment. Animals were sacrificed by decapitation after subdued calming, on day 17 of the study at ZT 4, during the circadian peak time period of daily fasting in these nocturnal animals, and blood and tissues samples were harvested for biochemical analyses. At sacrifice, blood samples were collected for analyses of plasma insulin, glucose, leptin, and TBARS, a circulating biomarker of systemic oxidative stress. Insulin resistance was calculated from fasting insulin and glucose values using the HOMA-IR analysis as fasting plasma glucose (mmol/L) times fasting serum insulin (mU/L) were divided by 22.5. The aorta was removed and rapidly frozen in liquid nitrogen for the subsequent protein level analyses of enzymatic biomarkers of vascular oxidative and nitrosative stress, including NADPH oxidase 4, iNOS, GTPCH 1, eNOS, and sGC. Livers were also quickly removed and frozen in liquid nitrogen for the subsequent determination of the total triglyceride level and epididymal and retroperitoneal fat pads were removed and weighed as an index of body fat store level. Similarly, the brain was rapidly removed and frozen on dry ice for analyses of MBH mRNA levels of neuropeptide (NPY, AgRP), glial cell neuronal physiology-regulating (Gfap, S100a10, Aqp4, Bmp4) and neuronal plasticity (Mef2c) genes involved in the regulation of peripheral metabolism.

### 5.3. Rationale for Assessing Vascular ROS/RNS Pathology from Isolated Aorta Tissue

The vascular tissue intracellular and interstitial levels of ROS and RNS are extremely short lived and thus very difficult to accurately “capture” from a frozen tissue sample as an assessment of tissue redox status. Therefore, in an effort to assess the in vivo chronic vascular oxidative and nitrosative stress status concurrent with measures of brain neurophysiology and peripheral metabolic tissue biochemistry, this study employed an evaluation of aortic protein levels of the major regulatory enzymes involved in setting the redox balance within the vascular tissue (NADPH oxidase 4, iNOS, GTPCH 1, eNOS, and sGC) as follows. Elevated NADPH oxidase 4 and iNOS levels are known to drive increased vascular oxidative and nitrosative stress and vascular damage in humans and in this SHR animal model of metabolic syndrome [26,27,28,29,30,31,32,33,34,35,36,37]. In conjunction with these alterations, an increase in eNOS levels is also observed in metabolic syndrome humans and SHR rats, though in an altered uncoupled enzymatic state resulting from the actions of increased ROS/RNS generated by elevated NADPH oxidase 4 and iNOS on the eNOS molecule and its cofactor tetrahydrobiopterin (BH_4_). Consequently, uncoupled eNOS produces less NO and more ROS and RNS, thus increasing further eNOS uncoupling and thus reducing the levels and activation of sGC, (an enzyme that translates the vascular health benefits of eNOS-derived NO). Such increased eNOS and decreased sGC changes are also observed in metabolic syndrome humans and SHR rats. Finally, as a correlative assessment of tissue redox balance, the GTPCH 1 level was determined since its levels are reduced upon the reversal of oxidative/nitrosative stress imposed by elevated levels of local hydrogen peroxide (generated by elevated NADPH oxidase 4 and iNOS), and also reduced by high levels of its end product, the eNOS cofactor BH_4_, responsible for maintaining a healthful, coupled eNOS structure and function (see Discussion for full details of these interactions).

### 5.4. Western Blot Analyses of Aortic Proteins

Frozen aortic tissue was homogenized in RIPA buffer with protease inhibitors (Santa Cruz Biotechnology, Dallas, TX, USA Cat# sc-24948) and centrifuged at 10,000× *g* for 10 min. Protein concentration in the supernatant was determined with BioRad (Hercules, CA, USA) protein assay kit Cat# 5000122, and the sample was heated to 100 °C in loading buffer (BioRad, Hercules, CA, USA Cat# 1610737). Twenty µg of total protein was loaded per lane onto a denaturing BioRad (Hercules, CA, USA) Criterion gel. Aorta proteins were quantified by Western blot analysis using BioRad (Hercules, CA, USA) Criterion and ChemiDoc systems following the manufacturers’ instructions. Criterion gradient tris-glycine precast gels, secondary antibodies, PVDF blotting membranes, molecular weight markers, and enhanced chemiluminescence (ECL) reagents were also purchased from BioRad (Hercules, CA, USA).

The eNOS (Cat# 610297) and iNOS (Cat# 610329) antibodies were purchased from BD Transduction Laboratories (San Jose, CA, USA); the NADPH Oxidase 4 antibody (Cat# ab60940-100) and sGC antibody (Cat # ab53084-100) were each purchased from Abcam (Cambridge, MA, USA); the GTPCH 1 antibody (Cat# sc-48510) and actin antibody (Cat# sc-47778) were purchased from Santa Cruz Biotechnology (Dallas, TX, USA).

Samples from the control- and bromocriptine-treated animals were loaded onto the same Criterion gel along with the molecular weight markers; band intensity was only compared within the samples loaded onto the same gel. A housekeeping protein (actin) was concurrently quantified on all gels, and the test protein amount was normalized to actin in the Western blot analysis. Protein bands were quantified with BioRad (Hercules, CA, USA) ImageLab 4.1 software and represented as a percent of control.

### 5.5. Quantitative PCR Analysis of MBH mRNAs

Frozen brains were sectioned (300 µm) using a cryostat (Model# 3050S, Leica, Buffalo Grove, IL, USA) at −20 °C, and 3 sequential brain slices through the level of the MBH (Bregma −2.30 to −3.30) were collected. The medial basal hypothalamus was punched from these brain slices using the third ventricle as reference (0.3 mm below the roof of the third ventricle and 1.5 mm lateral on both sides). The diameter of the punch area was 3 mm and included the VMH, arcuate, median eminence, and ventral DMH nuclei. Total RNA was isolated from MBH punched samples with Trizol Reagent (ThermoFisher, Waltham, MA, USA Cat# 15596026). Total RNA quantity and purity was determined by UV spectroscopy and cDNA was synthesized using SuperScript IV VILO MasterMix with ezDNAse (ThermoFisher, Waltham, MA, USA Cat# 11766050) using 2 µg of total RNA per sample. Real-time qPCR as performed with Taqman fast advance master mix (ThermoFisher, Waltham, MA, USA Cat# 4444964) on a AriaMX (Agilent, Santa Clara, CA, USA) qPCR instrument with the following primer/probe sets:

Gfap (glial fibrillary acidic protein) ThermoFisher, Waltham, MA, USA Cat# Rn00566603_m1
Mef2c (myocyte enhancer factor 2C) ThermoFisher, Waltham, MA, USA Cat# Rn01494040_m1
NPY (neuropeptide Y) ThermoFisher, Waltham, MA, USA Cat# Rn01410145_m1
AgRP (agouti related neuropeptide) ThermoFisher, Waltham, MA, USA Cat# Rn01431703_g1
S100a10 (S100 calcium binding protein A10) ThermoFisher, Waltham, MA, USA Cat# Rn01409218_m1
Aqp4 (aquaporin 4) ThermoFisher, Waltham, MA, USA Cat# Rn00563196_m1
Bmp4 (bone morphogenetic protein 4) ThermoFisher, Waltham, MA, USA Cat# Rn00432087_m1
The relative expression of genes was calculated using the 2^−ΔΔCq^ method.

RPLP0 (Ribosomal Protein Lateral Stalk Subunit P0) was quantified with Taqman assay ThermoFisher, Waltham, MA, USA Cat# Rn03302271_gH and used as the internal reference and the relative gene expression was calculated as a fold-change from control samples.

### 5.6. Biochemical Assays of Blood Samples and Analysis of Liver Lipid Content

Blood glucose concentrations were determined by a blood glucose meter (OneTouch Ultra, LifeScan, Inc, Milpitas, CA, USA). Plasma insulin and leptin were assayed by EIA using commercially available assay kits (Cat# 80-INSMR-CH01 and 22-LEPMS-E01 respectively, ALPCO Diagnostics, Salem, NH, USA). Plasma TBARS was determined with a TBARS assay kit (Cat# 700870, Cayman chemicals, Ann Arbor, MI, USA). Liver total triglyceride level was determined by homogenizing 100 mg of tissue in 1 mL of 5% NP-40, heating to 100 °C for 3 min, centrifuging at 10,000× *g* for 3 min, and assaying the subsequent supernatant for triglyceride content by a Triglyceride Determination Kit (Sigma-Aldrich, St. Louis, MO, USA, Cat # TR0100).

### 5.7. Blood Pressure (BP) Measurements

Systolic and diastolic BPs were measured on conscious animals with a tail-cuff volume pressure recording method (CODA-6 non-invasive blood pressure system, Kent Scientific Corp., Torrington, CT, USA) following manufacturer’s instructions. Several days before experimental recordings, rats were acclimated to the restraining cage and the tail cuff to minimize or reduce any stress influence on the readings. The BP measurements were performed before and after 14 days of treatment. The reported BP values per animal were the result of an average of 6–8 measurements.

### 5.8. Statistical Analysis

Statistical differences for the measured parameters between three groups were first analyzed for normality with the Shapiro–Wilk test and then evaluated using one-way ANOVA followed by pairwise post hoc (Holm–Sidak method) for normally distributed data or Kruskal–Wallis one-way analysis of variance on the ranks test followed by Dunn’s pairwise multiple comparison procedures test for non-normal data. For comparisons of study measures between only two test groups, Student’s *t* test was employed. All data are expressed as the mean ± SEM of the study population. A statistical value of *p* < 0.05 was considered statistically significant. Statistical analysis was performed with SigmaPlot Version 14.5 (Systat, San Jose, CA, USA).

## Data Availability

The data presented in this study will be made available on request from the corresponding author.
